# Inequity in imaging: Why it matters? A statement from the Equity, Diversity and Inclusion Subcommittee of the European Society of Radiology

**DOI:** 10.1186/s13244-025-02144-w

**Published:** 2025-11-27

**Authors:** Anagha P. Parkar, Amaka C. Offiah, Mihai-Alexandru Ene, Ioana-Andreea Gheonea, European Society of Radiology (ESR)

**Affiliations:** 1 Radiology Department, Haraldsplass Deaconess Hospital, Bergen, Norway; 2 Sports Traumatology and Arthroscopy Research Group (STAR Group), Department of Clinical Medicine, University of Bergen, Bergen, Norway; 3 Division of Clinical Medicine, Faculty of Health, University of Sheffield, Sheffield, UK; 4 Department of Radiology, Sheffield Children’s NHS Foundation Trust, Sheffield, UK; 5 Doctoral School, University of Medicine and Pharmacy of Craiova, Craiova, Romania; 6 Department of Medical Imaging, University of Medicine and Pharmacy of Craiova, Craiova, Romania; 7 European Society of Radiology, Vienna, Austria

**Keywords:** Equity, Diversity, Inclusivity, Radiology, Imaging (AI)

## Abstract

**Abstract:**

The ESR equity, diversity and inclusivity (EDI) subcommittee is a part of the Young ESR committee created in 2024. This statement paper is the first in our series regarding EDI and radiology. In this paper, we examine and discuss issues which have been studied and reported regarding the inequity of imaging services. Inequity is prevalent in radiology and imaging circles in Europe. The variations observed in women, ethnic, age, disabled, non-binary, and gender groups are examined, as well as the variations in radiology research and in artificial intelligence-related imaging. Radiology departments need to be aware of the existing variations in radiology services. They need to educate their personnel on the etiquette and interaction with diverse populations. There should be versatile equipment to serve patients with disabilities. Radiologists should be aware of the lack of evidence-based knowledge with regard to female and non-white populations. Regarding clinical AI, departments need to actively audit and check for possible biases in AI in clinical use.

**Critical relevance statement:**

Understanding how EDI affects patient care is vital to providing equitable service to all patients. Radiologists should be aware of the lack of evidence-based knowledge regarding female and non-white populations, and be sensibly critical of guidelines which lack proper evidence.

**Key Points:**

- The workflow of the department should be organised so that all patients are served equitably.
- Radiologists need to be aware of the lack of evidence-based knowledge about female and non-white populations, and be critical of guidelines which lack proper evidence.
- Regarding AI, radiologists must actively audit and check for possible biases in AI in clinical use.

**Graphical Abstract:**

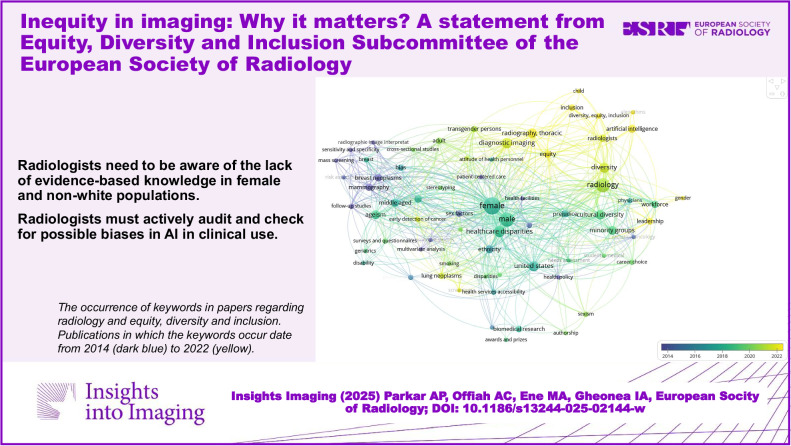

## Introduction

**Fig. 1:**
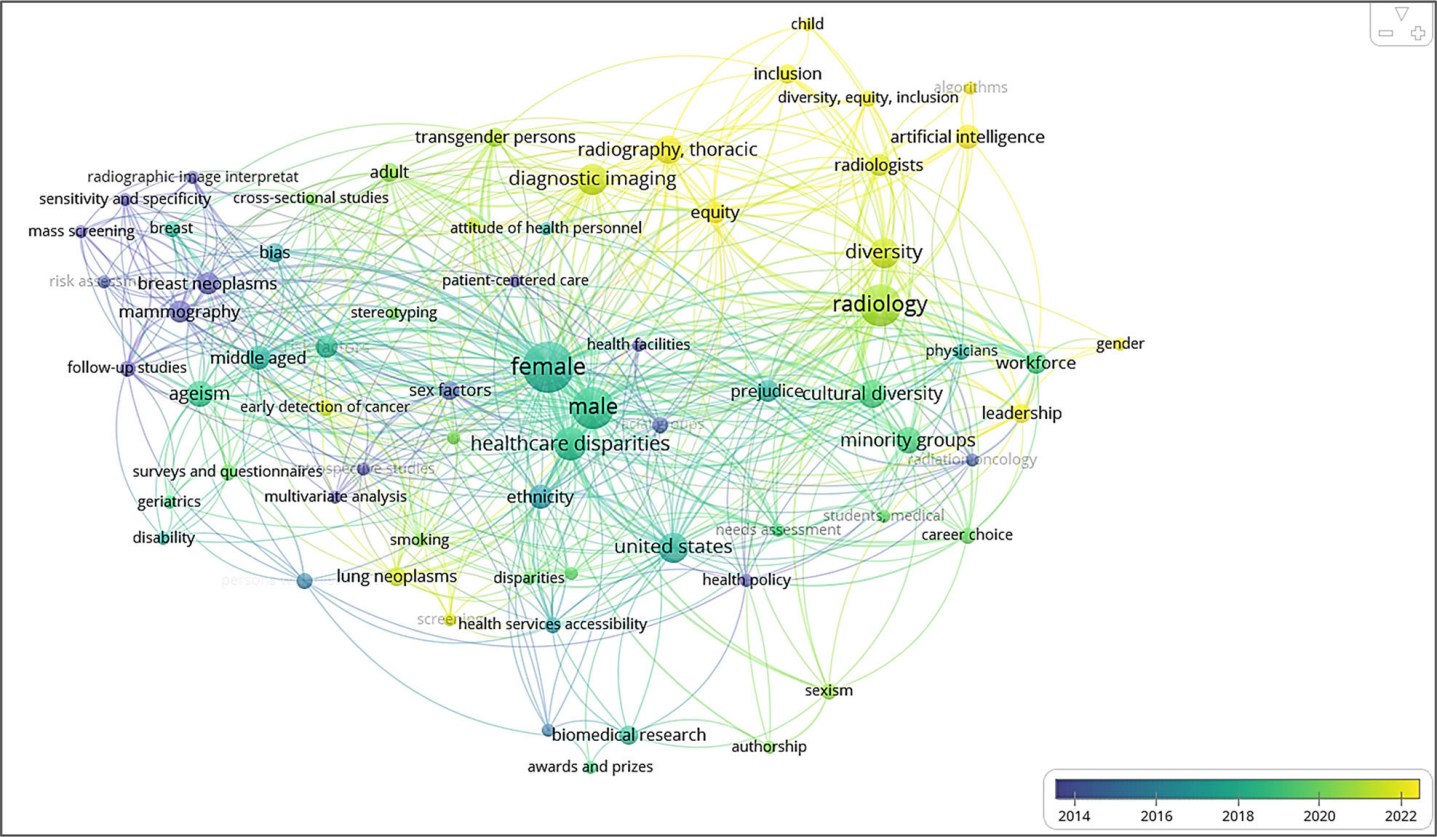
The image shows the occurrence of keywords in papers regarding radiology and equity, diversity and inclusion, sourced from a PubMed search on 21.01.25. Publications in which the keywords occur date from 2014 (dark blue) to 2022 (yellow). Image created in VOSviewer (www.vosviewer.com).

In recent years, many medical societies (including radiology societies) across the Western world have published papers discussing their position on the topic of Equity, Diversity and Inclusivity (EDI) in healthcare [1–7]. A quick search in PubMed for the term “radiology and EDI” exemplifies the most commonly used keywords and how they are linked (Fig. 1). Much of the initial focus has been on workforce issues, making the topic contentious [8–10].

The 75-year-old United Nations (UN) “Universal Declaration of Human Rights states that everyone has a right to equal access to public services [11]. As healthcare is a fundamental service to the public, EDI in healthcare (including radiology) is an extrapolation of this UN declaration.”

The UN “DEI glossary” has collated terminology from many valid sources [12]. Several important EDI terms have been unequivocally defined. In this paper, we adhere to the terminology defined by the UN.

The term *equity* mainly relates to being fair to all, but also includes addressing historical and present inequalities. The definition further mentions temporary use of special measures to alleviate systemic bias or discrimination, and that institutions can be considered equitable or inequitable, depending on their policies. It is also vital to understand that equity is not the same as equality, but is considered a means towards equality [12].

*Diversity* includes geographical distribution and sex and gender balance, as well as perspectives from ethnic, cultural, generational and persons with disabilities. It is suggested that embracing diversity can strengthen the performance of organisations [12]. In this paper, we have equated ethnicity with race, as both these terms are considered social constructs, and race is considered the broader term which subsumes ethnicity [12].

Finally, *inclusivity* is defined in terms of “inclusion” and is considered a dynamic state of belonging, in which diversity is valued, and which leads to a fair and results-based institution. An inclusive workplace culture is where employees feel safe, motivated and respected and are offered equitable opportunities.

One should take note that the definitions describe organisations and institutions and do not refer to individual responsibilities. All three definitions are internationally agreed upon [12].

The primary role for EDI in radiology is to promote fairness of services and adequate representation of diversity to optimise staff wellbeing so that they give optimal healthcare to patients.

**Fig. 2:**
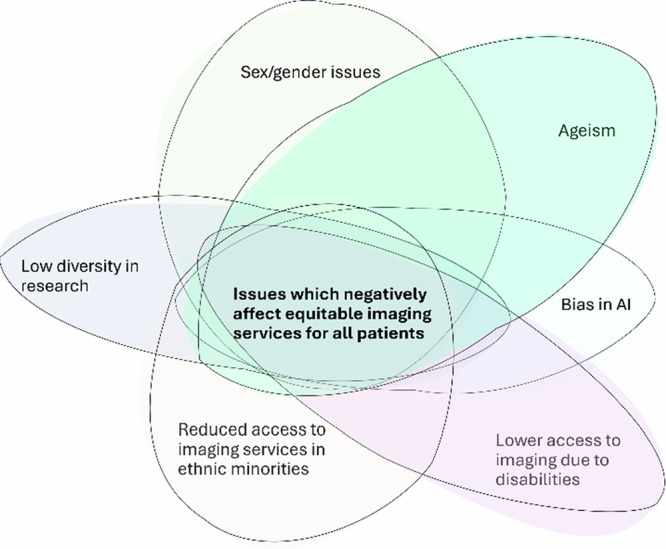
Graphical presentation of the issues which affect inequity in imaging services.

In this paper, we examine and discuss issues regarding inequity of imaging services, which is prevalent in radiology and imaging circles in Europe, to identify focus areas to improve the delivery of radiology services in the future (Fig. 2).

## Issues directly related to the delivery of patient care in imaging

EDI is essential for guaranteeing that all patients have access to equitable and bias-free imaging services. Although much more attention has been directed towards EDI as it relates to medical staff, it is also important to investigate the practical application of EDI tenets in patient care in the field of radiology. Any possible discrepancies impacting patient populations, including women, racial and ethnic minorities, the elderly, children, individuals with disabilities, and gendered minorities, should be identified and addressed (Table 1).

**Table 1:** Disparities in numbers

	Disparities in numbers:	Ref.
Women	More likely to experience delays in lung cancer diagnosis compared to men (4.2 months to diagnosis compared to 1.5 months in men).	[16]
	Less likely to get a COPD diagnosis even when presenting with the same symptoms, 42% compared to 58% in men.	[23]
Ethnicities	Non-whites are less likely to receive emergency imaging compared to whites; 41% vs 49%.	[27]
Ageism	A survey study revealed that 20% of elderly patients experienced prejudice related to their age.	[32]

A lot of the available data regarding EDI issues comes from the United States of America (USA), where patient identifiable data have long been systematically registered by healthcare administrators [13].

### Imaging in women

The variations in delivery of services to women were identified in the last century [14]. Mammography screening programmes were introduced in Europe from the 1980s onwards and have been successful, however, there are inexplicable delays in implementing improvements such as MRI screening for dense breasts [15]. Dense breast tissue not only reduces the accuracy of mammography but also elevates the risk of breast cancer, and MRI is superior to mammography for imaging dense breast tissue [16, 17]. In lung imaging, a study from Spain showed that women with a solitary nodule were more likely to receive follow-up, rather than progress to interventions, compared to men, which could lead to delayed final diagnosis [18, 19]. Recently, in pulmonary medicine, the sex variations in the respiratory physiology, asthma, interstitial lung disease, COPD and lung cancer, where most often women are underdiagnosed or experience delayed diagnosis, have been recognised and highlighted [20–24]. As current evidence shows that lung cancer screening is more efficient in females compared to men, there is a call to proactively recruit more female smokers into lung cancer screening schemes [25, 26].

### Ethnic minorities

A systematic review of > 200 studies has shown that ethnic minorities receive fewer and less appropriate imaging studies [27]. The group most affected are blacks, who are up to 21% less likely to receive imaging procedures and are less likely to undergo follow-up scans for incidental lung nodules [28, 29].

Another important aspect to consider is that image interpretation may be affected by implicit bias, potentially resulting in incorrect diagnoses or overlooked medical conditions in minority patients [30].

### Ageism

Ageism affects all age groups, and leads to categorisation and division, which leads to erosion of rights and discrimination [12, 31]. In radiology, older people may be under-referred for imaging investigations because of the false assumption that they will benefit less from certain medical procedures due to old age [32]. According to survey data, up to 20% of older patients reported encountering this prejudice during medical interactions [33]. Elderly patients or chronically ill (but not chronologically aged) patients are more prone to experience adverse effects after interventions, and frailty indices can help clinicians recognise patients at risk [34]. Imaging can aid immensely, as osteopenia and sarcopenia are useful age-related imaging biomarkers [34].

### Individuals with disabilities

Globally, more than one billion individuals live with a disability [35]. Visually obvious disabilities are often recognised, but the WHO definition of Individuals with disabilities.

considers disability as multidimensional; impairment in body function or structure, limitation in activity and/or restriction in participation. These patients frequently encounter barriers such as inaccessible radiology facilities, non-adjustable equipment, or insufficient support, hindering access to essential care [36]. For instance, studies show that women with chronic disabilities have reduced participation in breast cancer screening [37]. A national survey from 2010, in the US, reported that only 61% of women with disabilities aged 50–74 years underwent a mammogram in the preceding two years, in contrast to 75% of women without [38]. Other examples of patient groups often forgotten and not considered disabled are neurodiverse and obese patients. One should bear in mind that complications of severe obesity can lead to disability, and modifying imaging protocols to produce images of adequate quality ought to be actively implemented in radiology departments [39, 40]. In recent years, there has been an increased focus on how to improve imaging services for neurodiverse patients [41].

### Gender variations

A recent survey revealed that 25% of transgender and gender non-binary patients faced adverse interactions with radiology personnel, marked by disregard for personal comfort and failure to protect privacy [42]. Respectful, patient-centred communication implies the use of chosen names and preferred pronouns [43]. Conversely, transgender and non-binary patients are encouraged to disclose pertinent information to their healthcare providers [44]. According to another survey, fear of discrimination caused 28% of transgender patients to delay seeking medical attention, and 50% of them had to educate their healthcare providers regarding transgender care [45]. Radiology specialists need to stay informed about the distinct anatomical and physiological parameters in these patients [46]. Data on breast cancer screening in transmasculine transgender individuals is scant and based on expert opinion, transmasculine individuals should usually follow their birth sex’s screening recommendations for breast cancer, unless they have had a bilateral mastectomy. Transfeminine individuals often do not require breast cancer screening unless they have undergone a minimum of 5 years of gender-affirming hormone therapy [46, 47].

## Issues indirectly related to patient care

### Lack of diversity in radiology research

The importance of diversity in health research and, consequently, on patient outcomes is widely documented [48]. If healthcare to all patients is to be equitable, then it is clear that not only should research be conducted by diverse teams (so that appropriately diverse research questions are considered) [49], but also that research populations should be diverse [48]. Challenges to diversity, with regard to women and ethnic minorities, of research teams include inequitable awarding of research funds [50], lack of diversity of editorial boards [51], and low publication rates of research by minority groups [52–54].

Initiatives that might help redress some of these inequities have been suggested [53, 55, 56], with some authors sharing their means of incentivising physicians [57] and their experience of designing a diversity program for radiology [55].

These systemic biases in research teams, funding and editorial boards lead to the well-documented biases in research publications regarding authorship. For example, gender inequalities have been demonstrated in first (31.6% female) and last (19.3% female) authors in medical imaging journals (during the COVID-19 pandemic) [58]. In their review, Meshaka et al showed that of abstracts presented at international paediatric radiology conferences, only 1% were from low/lower middle income countries, with similar figures for presented articles that were published in the subsequent five to seven years [59].

The examples given above all relate to the radiology research workforce. A diverse research workforce positively impacts patient outcomes through collaboration, innovation and decreased implicit bias [60]. A diverse radiology research workforce should also lead to a diverse research participant pool, the importance of which is highlighted, for example, by the health disparities resulting from breast and lung screening programmes [61, 62].

### Bias in artificial intelligence (AI)-related imaging

As research endeavours increasingly focus on AI, it has become apparent that biases might potentially be exhibited by some algorithms, which could even be considered to contravene the principles of bioethics [63]. AI has been more widely taken up in medical imaging than in any other medical field. Biases in all phases of the AI development may lead to unwanted patient outcomes. Prejudices may relate to the research question being asked [64] or to data that is biased towards or against a specific race, sex, age, social class or other characteristic [65]. In the latter case, the developed AI tool will have higher performance for the group on which it was trained than for other groups. Reasons for these skewed datasets include sampling bias (e.g. paucity of images with normal findings, the so-called “negative set bias”), systemic bias and availability bias, all of which mean that the results of the algorithm cannot reliably be extended beyond the population on which it was trained [66]. This is important because it has been shown that incorrect AI causes radiologists to make incorrect decisions, for example, in mammography [67], in the detection of lung nodules [68], and interpretation of various chest radiographic findings [69]. To mitigate the risks and to improve the quality of AI research performance and reporting, a Checklist for Artificial Intelligence in Medical Imaging (CLAIM) was introduced in 2020 and updated in 2024 [70].

## Conclusion

Imaging is a constantly evolving field. We have presented the evidence for inequity in radiology in several fields, such as breast imaging, chest imaging and paediatric radiology. Further research is required to examine if these trends are also prevalent in high-volume radiology fields such as neuro, abdominal and musculoskeletal imaging. As healthcare personnel, we need to be aware of and actively implement the necessary changes in the workflow of the department in order to serve all patients equitably. This implies making investments in versatile equipment, educating personnel on disability etiquette and how to interact with diverse patient populations, and guaranteeing that buildings adhere to accessibility standards. As radiologists, we need to be aware of the lack of evidence-based knowledge with regard to female and non-white populations, as this can lead to delayed diagnosis. This implies being sensibly critical of guidelines which are lacking proper evidence, and addressing the same, when creating new guidelines. Finally, in the era of AI, we need to actively audit and check for possible biases in AI in clinical use. We must all strive to be active bystanders.
